# Inhibition of Ubiquitin-Specific Protease-13 Improves Behavioral Performance in Alpha-Synuclein Expressing Mice

**DOI:** 10.3390/ijms23158131

**Published:** 2022-07-23

**Authors:** Xiaoguang Liu, Kaluvu Balaraman, Ciarán C. Lynch, Michaeline Hebron, Priya Ketankumar Shah, Shicheng Hu, Max Stevenson, Christian Wolf, Charbel Moussa

**Affiliations:** 1 Department of Neurology, Translational Neurotherapeutics Program, Laboratory for Dementia and Parkinsonism, Lewy Body Dementia Association, Research Center of Excellence, Georgetown University Medical Center, Washington, DC 20057, USA; mlh88@georgetown.edu (M.H.); ps1199@georgetown.edu (P.K.S.); sh1737@georgetown.edu (S.H.); mes407@georgetown.edu (M.S.); 2 Department of Chemistry, Georgetown University & Medicinal Chemistry Shared Resource, Georgetown University Medical Center, Washington, DC 20057, USA; bk562@georgetown.edu (K.B.); ccl86@georgetown.edu (C.C.L.); cw27@georgetown.edu (C.W.)

**Keywords:** alpha-synuclein, USP13, BK50118-C, dopamine

## Abstract

Ubiquitin-Specific Protease-13 (USP13) promotes protein de-ubiquitination. USP13 levels are upregulated in post-mortem Parkinson’s disease, whereas USP13 knockdown via shRNA reduces alpha-synuclein levels in animal models. We studied the role of USP13 in knockout mice expressing lentiviral human alpha-synuclein and investigated the impact of a small molecule inhibitor of USP13, BK50118-C, on alpha-synuclein pathology and animal behavior. Alpha-synuclein was expressed unilaterally in substantia nigra (SN) of USP13 deficient mice that were treated with a daily intraperitoneal injection of 100 mg/kg BK50118-C or DMSO for four consecutive weeks, and behavioral and functional assays were performed. Wild-type USP13^+/+^ mice expressing lentiviral human alpha-synuclein showed motor and behavioral defects that were not seen in partially (USP13^+/−^) or completely (USP13^−/−^) deficient USP13 mice. BK50118-C displayed a wide and favorable therapeutic dose range in vivo. Treatment with BK50118-C significantly reduced ubiquitinated alpha-synuclein, increased dopamine levels, and improved motor and behavioral symptoms in wild-type (USP13^+/+^), but not USP13 deficient, mice. These data suggest that USP13 is critical to the neuropathology of alpha-synuclein, whereas a novel small molecule inhibitor of USP13 is a potential therapeutic agent of alpha-synucleinopathies.

## 1. Introduction

Ubiquitin-Specific Protease (USP)-13 is critical in the regulation of protein clearance mechanisms in models of Parkinson’s disease (PD) [1,2]. USP13 reduces the E3 ubiquitin ligase activity of parkin via de-ubiquitination [1,2], while USP13 knockdown increases parkin ubiquitination and activity and reduces alpha-synuclein levels [1,2]. USP13 may also be critical for the regulation of other E3 ubiquitin ligases that are implicated in PD pathology, including NEDD4 [3]. USP13 modulates the activity of ubiquitin-recognition protein Ufd1 to facilitate endoplasmic reticulum (ER)-associated degradation (ERAD) of misfolded proteins [4]. shRNAs targeting USP13 expression reduces alpha-synuclein via autophagy and or the proteasome in vivo and in vitro [1,5]. Knockdown via shRNA and pharmacological inhibition of USP13 increase clearance of ubiquitinited alpha-synuclein [1,2,5]. When taken together, these findings suggest a role of USP13 in alpha-synuclein-associated pathology.

De-ubiquitinases (DUBs), including USP13, regulate de-ubiquitination via handling the ubiquitin precursor or cleaving and removing the ubiquitin chain from the target protein. DUBs may also regulate de-ubiquitination indirectly via E3 ubiquitin ligases, including PD-linked parkin [1] or NEDD4 [6,7]. To date, more than 100 DUBs have been identified, which are divided into two major families, cysteine proteases and metalloproteases [8,9]. DUBs are sub-divided further into seven families according to their structure: ubiquitin C-terminal hydrolases (UCHs), ubiquitin-specific proteases (USPs), ovarian tumor proteases (OTUs), Machado–Josephin domain superfamily (MJD), MINDY family and ZUFSP family that belong to cysteine proteases, and JAB1/MPN/MOV34 metalloenzymes (JAMMs), which belongs to metalloproteases [8,9].

We synthesized novel small molecules [2], including BK50118-C, that are potent inhibitors of USP13 that effectively reduced alpha-synuclein levels and protected against neuronal death in models of alpha-synucleinopathies [2]. USP13 inhibition may prevent de-ubiquitination of alpha-synuclein and limit its aggregation via the promotion of autophagy and proteasome clearance [1,5]; thus, USP13 inhibitors may provide therapeutic agents in alpha-synucleinopathies [10]. We generated USP13 knockout mice and challenged them with lentiviral human alpha-synuclein to study the effects of USP13 on motor and behavioral symptoms and alpha-synuclein-associated pathology in the presence and absence of BK50118-C treatment.

Currently, there are three approaches to making PD animal models based on neurotoxins, transgenic animals, or viral vector-mediated gene delivery. Neurotoxin models induce acute effects but lack progression of underlying neurodegeneration of human PD pathology [11,12]. Most transgenic alpha-synuclein mouse models develop gradual alpha-synuclein pathology but fail to display clear dopaminergic cell loss and dopamine-dependent behavioral deficits. We directly deliver the alpha-synuclein gene to SN using a lentiviral vector. This model produces not only prolonged gradual alpha-synuclein aggregation and spreading pathology but also combined deterioration of the nigrostriatal system mimicking Parkinsonism [11,12]. Lentiviral vector allows long time alpha-synuclein expression and low immune response compared to other vectors [11,12]

## 2. Results

### 2.1. BK50118-C Increases Proteasome Activity and Reduces Alpha-Synculein

**Figure 1:**
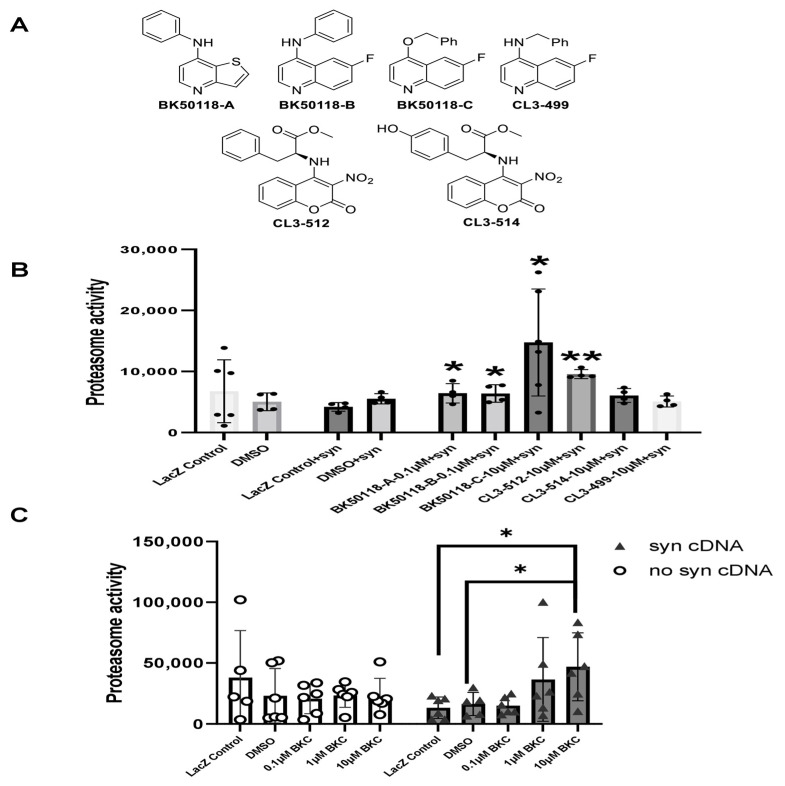
BK50118-C increases proteasome activity. Chemical structure of a small library of USP13 inhibitors (**A**). HEK 293ft cells transfected with alpha-synuclein cDNA or LacZ were treated with USP13 inhibitors. Measurement of 26S proteasome activity showed that (**B**) BK50118-A, BK50118-B, BK50118-C and CL3-512 increased proteasome activity (*n* = 4–6); (**C**) BK50118-C increased proteasome activity dose-dependently in alpha-synuclein stressed HEK 293ft cells but not in un-stressed cells (*n* = 5–6). The asterisk indicates statistically significant difference. One-way ANOVA, * *p* < 0.05, ** *p* < 0.01. Mean ± SD. The black dot ● in subfigure (**B**) represents each individual data.

A small library of USP13 inhibitors (Figure 1A) bearing 6-fluoroquinoline, thieno [3,2-*b*] pyridine, and 3-nitrocoumarin backbones were synthesized as previously reported [2]. BK50118-C was shown to be a lead candidate based on its pharmacodynamics profile [2]. Here we investigated the effects of BK50118-C and other USP13 inhibitors on proteasome activity and alpha-synuclein-stressed HEK293ft cells. Measurement of the activity of the 26S proteasome demonstrated that BK50118-A, BK50118-B, and BK50118-C significantly increased proteasome activity at 0.1 µM or 10 µM, and CL3-512 significantly increased proteasome activity at 10 µM (Figure 1B, *n* = 4–6). Remarkably, BK50118-C increased proteasome activity dose-dependently in alpha-synuclein-stressed cells but not in un-stressed cells (Figure 1C, *n* = 5–6). 

### 2.2. Determination of a Maximal Tolerated Dose and Tissue Toxicity

We performed a dose escalation study in 9–10 months old wild-type C57BL/6N mice in order to find a maximally tolerated dose and tissue toxicity. Dose escalation study (Supplementary Figure S1A, *n* = 4 per group, 2:2 male and female) showed no visible adverse effects in mice treated with BK50118-C up to 170 mg/kg IP daily. This was confirmed by autopsy. The treatment stopped after a 7-day administration of 170 mg/kg IP due to irritation at the injection site. Therefore, 170 mg/kg IP may be near or at the maximally tolerated dose in mice. We also verified tissue cell death via caspase-3 activity assay that showed no differences in drug-treated mouse tissues, including liver, brain, kidney, lung, spleen, heart, and small intestine, compared to control mice (Figure S1B, *n* = 4 per group).

### 2.3. Lentiviral Expression of Human Alpha-Synuclein Induces Motor and Behavioral Symptoms in USP13^+/+^ but Not in USP13^+/−^ or USP13^−/−^ Mice

**Figure 2:**
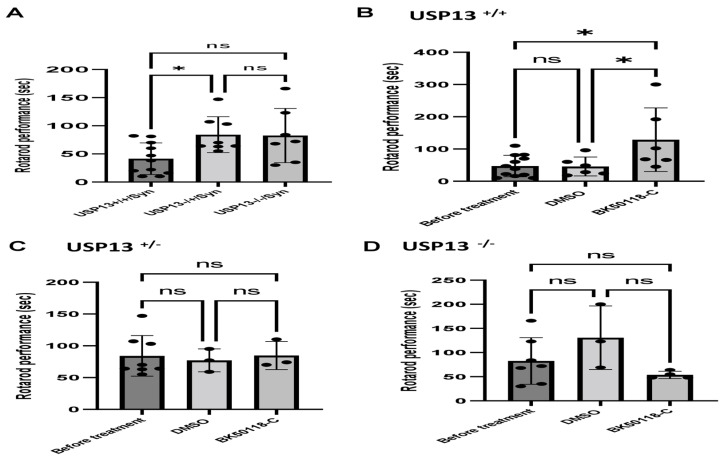
Rotarod behavior test in mice. Rotarod test showed that lentiviral expression of human alpha-synuclein induced motor and behavioral symptoms in wild-type (USP^+/+^) but not in partially (USP^+/−^) or completely (USP13^−/−^) mice (**A**). BK50118-C improves motor and behavioral symptoms in wild-type USP^+/+^ (**B**), but not in USP^+/−^ (**C**), or USP13^−/−^ (**D**) mice. The asterisk indicates statistically significant difference. One-way ANOVA. * *p* < 0.05. *n* = 3–12. Mean ± SD. The black dot ● represents each individual data. ns: no significance.

**Figure 3:**
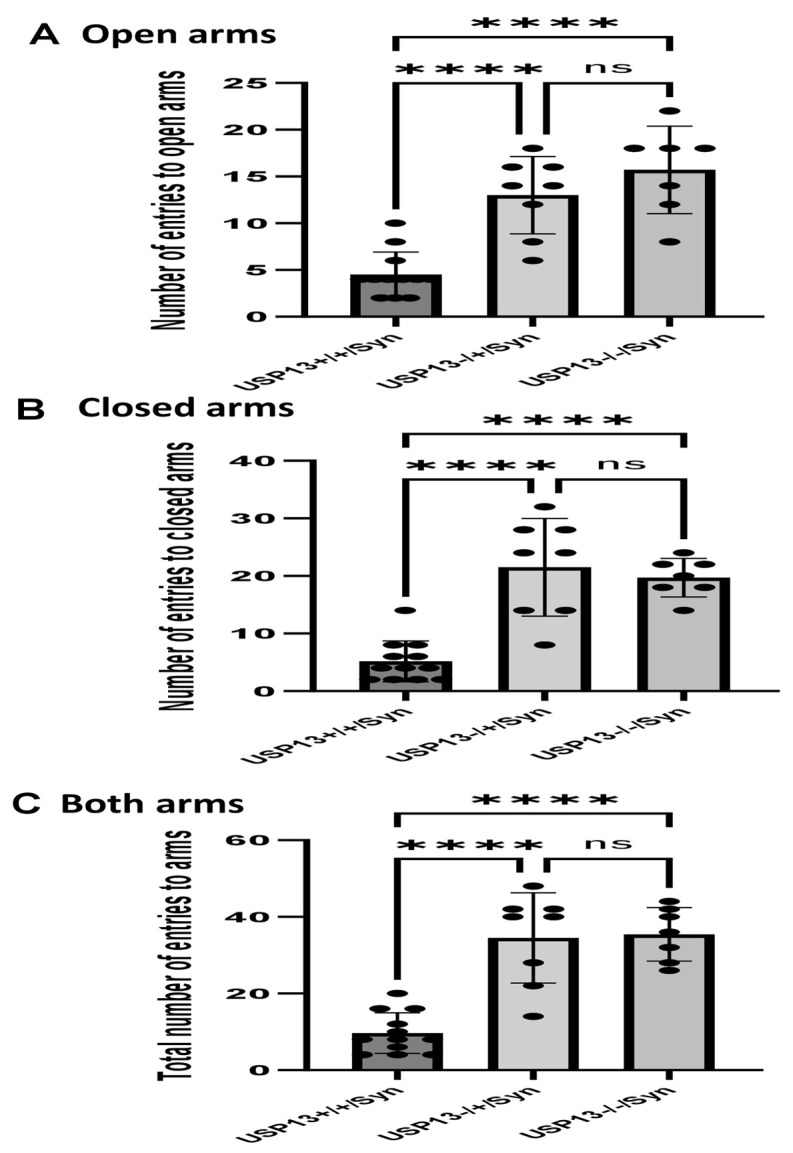
Human alpha-synuclein expression in SN of USP^+/+^ mice led to reduced motor activities in elevated plus maze test. Two weeks after human alpha-synuclein injection into right SN, elevated plus maze showed that number of entries to open arms (**A**) number of entries to closed arms (**B**) and total number of entries to arms (**C**) reduced in USP^+/+^ mice compared to USP^+/−^ or USP13^−/−^ mice. The asterisk indicates statistically significant difference. One-way ANOVA. **** *p* < 0.0001. *n* = 7–12 per group. Mean ± SD. The black dot ● represents each individual data. ns: no significance.

**Figure 4:**
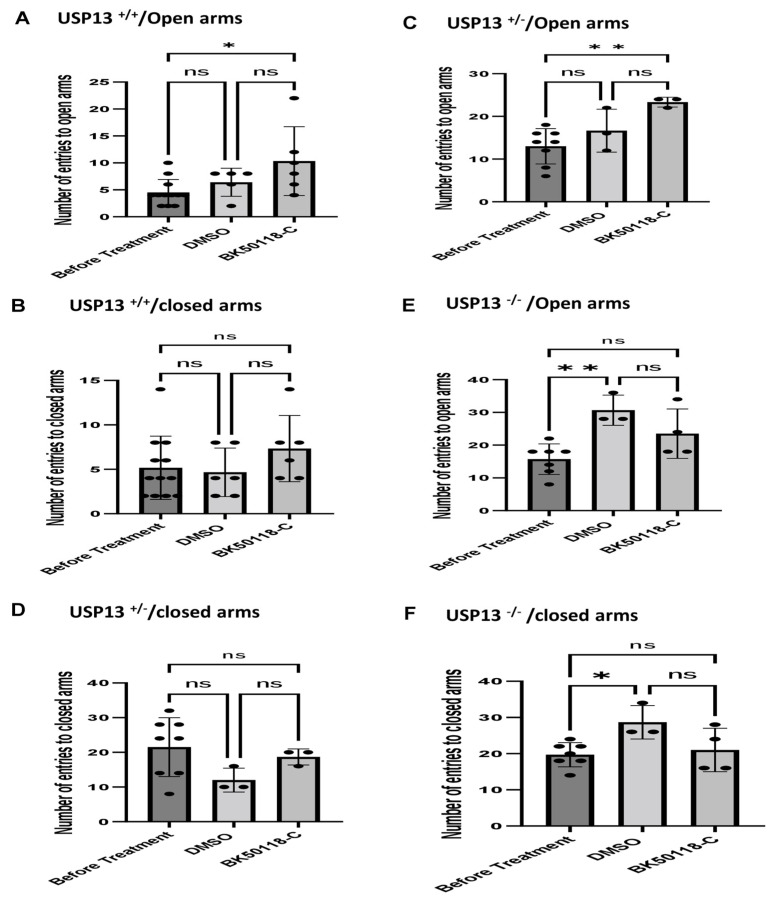
BK50118-C reduces anxiety in USP^+/+^ and USP^+/−^ but not in USP13^−/−^ mice stressed with alpha-synuclein. Elevated plus maze test showed that BK50118-C increased number of entries to open arms in (**A**) USP+/+ and (**C**) USP^+/−^ mice, but not in number of entries to closed arms (**B**,**D**). BK50118-C had no significant effects on the number of entries to either open (**E**) or closed (**F**) arms in USP13^−/−^ mice. The asterisk indicates statistically significant difference. One-way ANOVA, non parametric test. * *p* < 0.05, ** *p* < 0.01. *n* = 3–12 per group. Mean ± SD. The black dot ● represents each individual data. ns: no significance.

We previously showed that BK50118-C reduces alpha-synuclein levels and improves neuronal survival in transgenic mice that carry the human A53T mutation of alpha-synuclein (A53T) [2]. To ascertain that USP13 is a specific target of BK50118-C, we propagated a colony of USP13 deficient mice (C57BL/6N-Usp13^tm1b(EUCOMM)Hmgu^/WtsiH) that included partial (USP13^+/−^) or complete deletion (USP13^−/−^) of USP13 in comparison with wild-type littermates (USP13^+/+^). Male and female mice (6–7 months of age) were injected with lentiviral alpha-synuclein directly into the right *Substantia Nigra* (SN) via stereotaxic surgery, as we previously explained [1]. Three weeks after surgery, mice were treated with a daily IP injection of 100 mg/kg BK50118-C for 4 consecutive weeks. Unilaterally human alpha-synuclein expression in the right SN led to poor motor performance in USP^+/+^ (WT), compared to USP^+/−^ or USP13^−/−^ mice (Figure 2A, *n* = 7–12 per group) via Rotarod. Interestingly, BK50118-C treatment led to a significant motor improvement in USP13^+/+^ (Figure 2B, *n* = 6–12) compared to DMSO treated mice, but these effects were not observed in either USP^+/−^ (Figure 2C, *n* = 3–8) or USP13^−/−^ (Figure 2D, *n* = 3–7) mice. Further behavioral studies of USP13 via elevated plus maze also showed that human alpha-synuclein expression led to a reduction in the number of entries to both the open (Figure 3A, *n* = 7–12 per group) and closed arms (Figure 3B, *n* = 7–12 per group), and the total number of entries to both arms (Figure 3C, *n* = 7–12 per group) in USP^+/+^ compared to USP13^+/−^ or USP13^−/−^ mice. Similarly, the elevated plus maze showed that BK50118-C increased the number of entries to the open arms in USP^+/+^ (Figure 4A, *n* = 6–12) and USP^+/−^ (Figure 4C, *n* = 3–8) mice, but not in the number of entries to closed arms (Figure 4B,D, *n* = 3–12). BK50118-C had no significant effects on the number of entries to either open (Figure 4E, *n* = 3–12) or closed (Figure 4F, *n* = 3–12) arms in USP13^−/−^ mice.

### 2.4. BK50118-C Lowers Alpha-Synuclein in USP13^+/+^ but Not in USP13^+/−^ or USP13^−/−^ Mice

**Figure 5:**
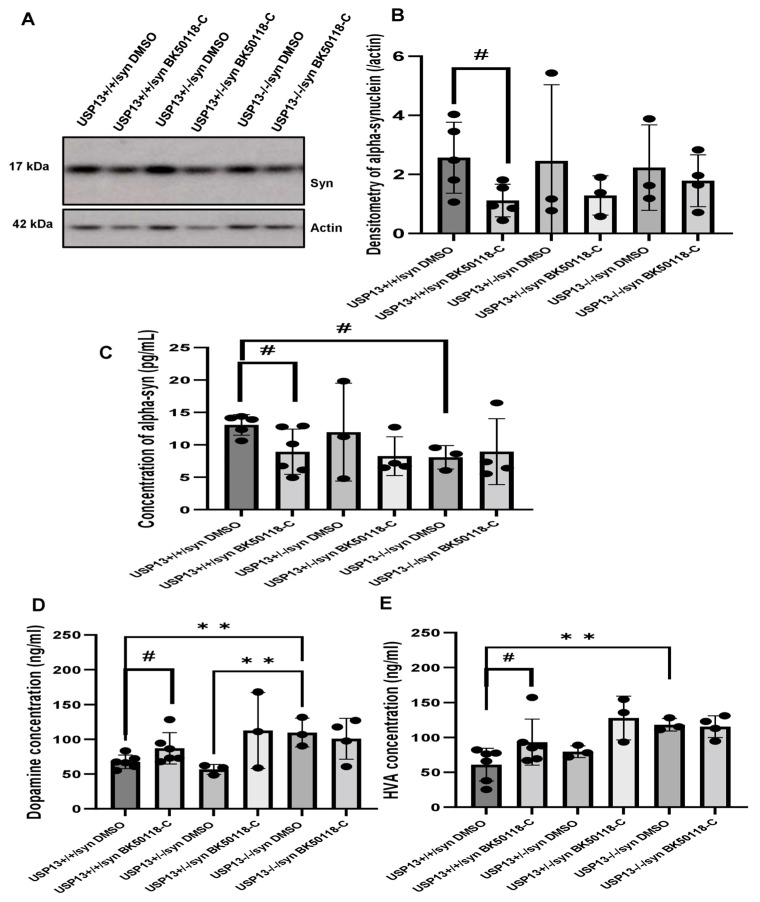
BK50118-C reduces alpha-synuclein and protects against dopamine loss in USP13^+/+^, but not USP^+/−^ or USP13^−/−^ brain. WB of STEN (soluble) midbrain extracts of (**A**) USP13^+/+^, USP^+/−^ and USP13^−/−^ mice, which were injected with lentiviral alpha-synuclein into SN and treated with BK50118-C or DMSO showing alpha-synuclein (17 kD) level relative to actin (42 kD) on 4–12% Criterion™ XT Bis-Tris Protein Gel, and (**B**) alpha-synuclein densitometry. ELISA measuring levels of (**C**) human alpha-synuclein (**D**) dopamine, and (**E**) HVA in same brain extracts. The asterisk indicates statistically significant difference. One-way ANOVA ** *p* < 0.01. Two tailed Student’s *t* test, and non parametric tests # *p* < 0.05. *n* = 3–6 per group. Mean ± SD. The black dot ● represents each individual data.

Western blot (WB) analysis of midbrain extracts showed that BK50118-C significantly reduced the level of alpha-synuclein (Figure 5A,B, *n* = 3–6 per group) in USP13^+/+^ but not in USP13^+/−^ or USP13^−/−^ mice. ELISA also demonstrated that BK50118-C significantly reduced the level of alpha-synuclein (Figure 5C, *n* = 3–6 per group) in USP13^+/+^ but not in USP13^+/−^ or USP13^−/−^ mice. Compared to USP13^+/+^ mice, the level of alpha-synuclein in the brain significantly decreased in USP13^−/−^ mice (Figure 5C, *n* = 3–6 per group).

### 2.5. BK50118-C Protects against Dopamine Loss in USP13^+/+^ but Not in USP13^+/−^ or USP13^−/−^ Mice

Measurement of dopamine and its metabolite homovanillic acid (HVA) levels in midbrain tissue extracts showed higher levels of dopamine and HVA in USP13^−/−^ compared to USP13^+/+^ (Figure 5D,E, *n* = 3–6 per group). BK50118-C treatment significantly increased dopamine levels in USP13^+/+^ (Figure 5D, *n* = 3–6 per group) and USP^+/−^ mice (Figure 5E, *n* = 3–6 per group). BK50118-C also significantly increased HVA levels in USP13^+/+^ (Figure 5D, *n* = 3–6 per group) and USP^+/−^ mice (Figure 5E, *n* = 3–6 per group). These results suggest that BK50118-C protects against dopamine loss.

### 2.6. BK50118-C Lowers the Level of Ubiquitinited Alpha-Synuclein and Increases Autophagy

Co-immunoprecipitation (CO-IP) of ubiquitin (Figure S2A, *n* = 3–6) or alpha-synuclein (Figure S2B, *n* = 3–6) from midbrain lysates of USP13^+/+^, USP13^+/−^ or USP13^−/−^ mice followed by WB shows that BK50118-C increased ubiquitinited protein levels and decreased alpha-synuclein levels, especially in USP13^+/+^ mice. These data indicate that BK50118-C increases alpha-synuclein ubiquitination.

Autophagy is another major pathway to degrade misfolded proteins. LC3-I conversion to LC3-II by lipidation is a marker of autophagosome formation, while LC3-II reduction may be indicative of autophagosome clearance. WB of midbrain extracts showed that BK50118-C significantly reduced the ratio of LC3-II relative to LC3-I in USP13^(+/+)^ mice but not in USP13^+/−^ and USP13^−/−^ mice, suggesting clearance of autophagosome in USP13^(+/+)^ mice but not in USP13^+/−^ and USP13^−/−^ mice (Figure S2C,D, *n* = 3–6).

## 3. Discussion

The present data show that USP13 plays a critical role in alpha-synuclein-associated pathology. We previously showed that USP13 knockdown via shRNAs could reduce neurotoxic proteins, including alpha-synuclein, via autophagy and or the proteasome in vivo and in vitro [1,5], consistent with our data. We also demonstrated that pharmacological inhibition of USP13 increases clearance of ubiquitinited alpha-synuclein in A53T mice [1,2,5], in agreement with our results. Here we show that expression of human alpha-synuclein in the SN may lead to motor and behavioral defects in USP13^+/+^ but not in USP13^+/−^ or USP13^−/−^ mice. USP13^+/+^ mice displayed both motor and behavioral symptoms, suggesting increased levels of anxiety when challenged with human alpha-synuclein. However, partial or complete deletion of USP13 did not appear to lead to behavioral and motor defects. Furthermore, dopamine loss was evident in USP13^+/+^ mice, and this loss of dopamine was not seen in USP13 deficient mice. Dementia with Lewy Bodies (DLB) and PD is characterized by the accumulation of misfolded alpha-synuclein, which forms Lewy body (LB) inclusions [13,14]. We previously demonstrated that the expression of USP13 is elevated in post-mortem brains with neurodegeneration, including the nigrostriatum of PD patients [5,15]. It is possible that the accumulation of alpha-synuclein induces oxidative stress and affects USP13 levels of activity, as an expression of the usp13 gene increases under stress conditions, including oxidative stress [16]. These data are congruent with our previous findings that USP13 knockdown via shRNA regulates the E3 ubiquitin ligase activity of autosomal recessive PD-linked parkin and promotes ubiquitination of alpha-synuclein, leading to enhanced clearance via autophagy and the proteasome [1,5]. USP13 is widely distributed in human tissue, including the cytosol and nucleoplasm [17,18], and its main role is protein de-ubiquitination via cleavage of ubiquitin chains [17]. Our data also showed via co-immunoprecipitation that ubiquitin-linked alpha-synuclein is reduced in USP13 deficient mice, suggesting that USP13 de-ubiquitinates alpha-synuclein and BK50118-C prevents USP13 effects. The concurrent reduction of alpha-synuclein and improvement of dopamine metabolism are consistent with previous findings showing that aggregation of alpha-synuclein impairs dopamine transmission, but the elimination of misfolded alpha-synuclein improves dopamine transmission [19,20].

We previously demonstrated that our small molecule USP inhibitors are brain-penetrant [2], and they regulate (de)-ubiquitination of neurotoxic proteins to improve neurodegenerative pathology in mouse models of alpha-synucleinopathy [2]. We further investigated the role of BK50118-C and its therapeutic potential in USP13 deficient mice. We observed that our lead compound BK50118-C, which displays an optimal IC_50_ (0.42 nM) towards USP13 [2], also showed maximal activation of the 20S proteasome as well as LC3-II reduction, suggesting that the effects of this compound on reduction of ubiquitinated alpha-synuclein are via proteasome and autophagy. Partial or complete deletion of USP13 did not appear to lead to behavioral and motor defects, and administration of BK50118-C did not seem to affect behavior. Nonetheless, alpha-synuclein accumulation, dopamine loss, and motor and behavioral defects were attenuated in USP13^+/+^ treated with BK50118-C, suggesting that this agent may primarily target USP13. We previously showed that USP13 knockdown via shRNAs could reduce alpha-synuclein via autophagy [1,5]. A wide therapeutic range of BK50118-C (up to 1–170 mg/kg) was established in mice, and cell death assays in many tissues demonstrated that this dose range is safe, suggesting that potentially higher doses than 100 mg/kg used in the current experiments could be administered. Overall, BK50118-C seems to be a potent USP13 inhibitor that may prevent alpha-synuclein de-ubiquitination, and this study suggests that USP13 is a therapeutic target in PD and DLB [10].

Structurally, USP13, also called isopeptidase T-3 [21], contains one USP domain and four zinc finger ubiquitin binding (ZnF-UBP) domains, including one catalytic site, two ubiquitin-associated (UBA) domains, and one ZnF domain [22]. The tandem UBA domain interacts with ubiquitin and acts as the receptor for USP13 catalytic activity [17]. The UBA and ubiquitin interacting motif (UIM) domains of USP13 promote substrate and enzyme binding, and the ZnF domain facilitates enzyme hydrolysis [17]. USP13 and USP5 are homologous with similar structures but different functions, with the activity of USP13 being lower than that of USP5 [23]. USP13 also lacks the ability to hydrolyze free polyubiquitin chains and mainly regulates the expression of specific substrates [22]. USP13 catalyzes the hydrolysis of K63-linked ubiquitin chains attached to target proteins, which may result in proteasome and autophagy degradation [17,23]. Taken together, the variety and functions of different DUBs, including USP13 and USP5, suggest that a comprehensive screening for all DUBs is required to determine whether BK50118-C targets any other USPs. More relevant to movement disorders, USP8 may control alpha-synuclein ubiquitination and clearance via the proteasome [24]. USP19 is also associated with polyglutamine-expanded (polyQ) diseases, including Huntington’s disease (HD) and spinocerebellar ataxia [25,26,27]. USP33 de-ubiquitinates parkin and antagonizes its role in mitophagy [28]. PTEN-induced kinase 1 (PINK1) activates parkin to degrade depolarized mitochondria [29,30,31] and promotes misfolded protein clearance [31]. When taken together, these findings suggest that DUBs, including USP13, may play a critical role in the pathology of several movement disorders.

Different inhibitors of USP13 are also under investigation. Spautin-1 inhibits USP13 expression, but it does not cross the BBB, and it inhibits both USP10 and USP13 and affects the proteasome and autophagy at a much higher concentration than BK50118-C [32]. USP13 appears to be a major regulator of autophagy via interaction with the Beclin-1-parkin protein complex and autophagosome maturation [15,33,34]. The E3 ubiquitin ligase NEDD4/NEDD4-1 activates class III phosphoinositide 3-kinase (PIK3C3) to form a complex with the vacuolar protein sorting 34 (VPS34) via ubiquitination, whereas USP13 decreases ubiquitination of PIK3C3 and reduces autophagy [6,7]. Knockout of either NEDD4-1 or USP13 increases ubiquitination and degradation of VPS34, thus attenuating autophagosome formation [6]. USP13 also interacts with the ubiquitin-recognition protein Ufd1 and facilitates endoplasmic reticulum (ER)-associated degradation (ERAD) of misfolded proteins [4]. Many other USPs, including USP20 and USP30, regulate auto-lysosome formation [35].

## 4. Materials and Methods

### 4.1. Structures and Synthesis of USP13 Inhibitors

BK50118-A, BK50118-B, BK50118-C, CL3-499, CL3-512 and CL3-514 were prepared from commercially available 7-chlorothieno [3,2-*b*] pyridine, 4-chloro-6-fluoroquinoline and 4-chloro-3-nitro-2*H*-chromen-2-one as we previously reported [2].

### 4.2. Cell Lines, Transfection, and Treatment

Human HEK 293ft cells were procured from the Tissue Culture and Biobanking Shared Resource at Georgetown University Lombardi Comprehensive Cancer Center. HEK 293ft cells were cultured in DMEM with 10% FBS and 1% Gibco^TM^ PenStre(ThermoFisher, Rockford, IL, USA, 15140122). SH-SY5Y cells were cultured in DMEM with Ham’s F12 (1:1) (ThermoFisher, Rockford, IL, USA, 11765054) with 10% FBS, 1% PenStrep, and 1% L-glutamine. Cells were plated at a density to reach 70–80% confluence at the beginning of every experiment.

For 26S proteasome assay, HEK 293ft cells were transiently transfected for 24 h with human wild-type alpha-synuclein or lacZ vectors using Fugene HD transfection reagent (Promega, Madison, WI, USA, E2311) according to manufacturer’s protocol. Cells were treated with 0.1 µM or 1 µM, or 10 µM of each novel compound (BK50118-A, BK50118-B, BK50118-C, CL3-499, CL3-512, and CL3-514) dissolved in DMSO for 5 h. Cells were then harvested on ice by removing culture medium and adding 0.2 mL 1× sodium-tris, EDTA, NP-40 (STEN) buffer (50 mM Tris (pH 7.6), 150 mM NaCl, 2 mM EDTA, 0.2 % NP-40, 0.2 % with Halt protease and phosphatase inhibitor solution (ThermoFisher, 78446). Cells were homogenized and extracted by centrifuging at 10,000 g for 20 min at 4 °C. The supernatants were used for proteasome assay.

### 4.3. Proteasome Assay

Cell extracts (25 µg) were incubated with 50 µL of Proteasome-Glo™ Reagent containing Z-LRR-Glo™ Substrate coupled to luciferin (Proteasome-Glo™Tripsin-like assay, Cat No: G8631, Promega Corporation, Madison, WI, USA) for 30 min in dark. Proteasome-induced substrate cleavage generates a “glow-type” luminescent signal produced by the luciferase reaction. Record luminescence signal with a plate-reading luminometer. As a control, 0.25 mM of proteasome inhibitor lactacystin was applied for 5 h prior to the beginning of proteasome activity assays.

### 4.4. Caspase-3 Activity Assay

Caspase-3 activity was measured using Caspase-3 Colorimetric Activity Assay Kit, DEVD (Cat No: APT165, Millipore Sigma, Burlington, MA, USA). The assay is based on spectrophotometric detection of the chromophore *p*-nitroaniline (pNA) after cleavage from the labeled substrate DEVD-pNA. The free pNA can be quantified using spectrophotometer at 405 nm for caspase-3 activity measurement.

### 4.5. Determination of Maximal Tolerated Dose and Tissue Toxicity

In order to determine the maximally tolerated dose of BK50118-C and the tissue toxicity, a dose escalation study was performed. Male and female C57B/L6 (weighing 30 ± 5 g) mice were treated with BK50118-C starting from daily dose of 10 mg/kg intraperitoneal (i.p) for 7 days and then gradually increased the dosage to 50, 80,110, 140, and up to 170 mg/kg i.p injection. Each dosage was a 7 day-treatment. The treatment stopped due to peritonitis at injection site, but all mice survived, and no other abnormal behavior sign was observed.

Mice were then sacrificed under ketamine/xylazine anesthesia. Heart, lung, liver, brain, small intestine, spleen, and kidney were removed, homogenized, and extracted. The Caspase-3 activity was measured according to manufacturer’s protocols. To measure caspase-3 activity, we used caspase-3 colorimetric activity assay kit (Cat No: APT165, Millipore Sigma, Burlington, MA, USA) on tissue extracts and DEVD-pNA substrate. The absorbance was read according to manufacturer’s protocol.

### 4.6. Transgenic Mice, Surgery and Treatment

Male and female (6–7 months old of age) wild-type USP13^+/+^ and transgenic littermates, including partial (USP13^+/−^) or complete (USP13^−/−^) USP13 deletion were generated using C57BL/6N-^Usp13<tm1b^(EUCOMM)Hmgu>/WtsiH, purchased from MRC Harwell, Harwell Campus, Oxfordshire, OX11 0RD, Didcot, UK). Stereotaxic surgeries were performed to inject 1 × 10^9^ moi lentiviral alpha-synuclein into the right SN as we previously described [15]. Three weeks post-surgery, half of the mice were treated with intraperitoneal (IP) injection of 100 mg/kg BK50118-C and the other half with 3 µL DMSO every day for 4 consecutive weeks. All animals were sacrificed 4–5 weeks post-therapy. Rotarod test and elevated plus maze test were performed on all animals before sacrifice. To verify transgene expression, we routinely perform polymerase chain reaction (PCR) with the primers that were used to clone USP13 into lentiviral vectors. Animals were age- and sex-matched in their respective treatment cohorts. All experiments were conducted in full compliance with the recommendations of Georgetown University Animal Care and Use Committee.

### 4.7. Behavior Tests

***Rotarod test:*** Mice were placed on an accelerating rotarod (Columbus Instruments, Columbus, OH, USA) equipped with individual timers for each mouse. Mice were trained to stay on the rod at a constant 5 rpm rotation for at least 5 min, then the speed will gradually increase by 0.2 rpm/minute, and the latency to fall was measured. Mice have been trained for 3 consecutive days before the final measurement.

***Elevated plus maze test:*** This test uses an elevated, plus-shaped (+) apparatus with two open and two enclosed arms. The behavioral model is based on the general aversion of rodents to open spaces and measures anxiety in general. For testing, mice were placed individually in the center of the maze; the number of entries to open arms, the number of entries to closed arms, and total number of entries to both open and closed arms during 10-min period were recorded by trained and blinded observers.

### 4.8. Western Blot Analysis

In order to extract the soluble proteins from mouse midbrain lysates, tissues were isolated and homogenized in 1× STEN buffer (50 mM Tris (pH 7.6), 150 mM NaCl, 2 mM EDTA, 0.2% NP-40, 0.2% BSA, 20 mM PMSF and protease cocktail inhibitor), centrifuged at 10,000× *g* for 20 min at 4 °C and the supernatant containing the soluble protein fraction was collected. We performed protein quantification assays and normalized protein levels in the homogenized tissue samples prior to the WB, ELISA, and Dopamine/HVA assays. We used an equal number of proteins for the assays and also quantified using standard graphs.

Extracts were analyzed by western blot (WB) on 4–12% Criterion™ XT Bis-Tris Protein Gel (Bio-rad, #3450125). Beta-actin (β-actin) was probed (1:3000) with monoclonal antibody (Emdmillipore, MAB1501R). Human alpha-synuclein was probed (1:2000) with monoclonal antibody (Thermo Fisher, AHB0261, Rockford, IL, USA). Ubiquitin was probed (1:5000) with polyclonal antibody (Thermo Fisher, PA3-16717, Rockford, IL, USA). LC3-I and LC3-II were probed with polyclonal antibody (Thermo Fisher, PA1-16931, Rockford, IL, USA). WBs were quantified by densitometry using Quantity One 4.6.3 software (Bio Rad, Hercules, CA, USA) and Image J.

### 4.9. Enzyme-Linked Immunosorbent Assay (ELISA)

Human alpha-synuclein ELISA (Biolegend, Cat # 844101, San Diego, CA, USA) was performed on brain tissue extracts as described above according to manufacturers’ protocol.

### 4.10. Co-Immunoprecipitation

Mouse brain tissues were homogenized in 1× STEN buffer, and the soluble fraction was isolated as indicated above. The lysates were pre-cleaned with immobilized recombinant protein A/G agarose (Santa Cruz, sc-2003, Dallas, TX, USA) and centrifuged at 2500× *g* for 1 min at 4 °C. The supernatant was recovered and quantified by protein assay, and a total of 300 µg protein was incubated overnight at 4 °C with primary anti-alpha-synuclein (1:100) (Novus, NBP1-05194, Centennial, CO, USA) or anti-ubiquitin (1:100) (Thermo Fisher, PA3–16717, Rockford, IL, USA) antibodies in the presence of sepharose G and an IgG control with primary antibodies. The immunoprecipitates were collected by centrifugation at 2500× *g* for 3 min at 4 °C, washed 5 times in PBS, with spins of 3 min, 2500× *g* using detergent-free buffer for the last washing step, and the proteins were eluted according to Pierce instructions (Pierce #20365, Rockford, IL, USA). After IP, the samples were size-fractionated on 4–12% Criterion™ XT Bis-Tris Protein Gel and transferred onto 0.45 µm nitrocellulose membranes. The primary antibodies used for WB analysis of alpha-synuclein and ubiquitin were the same as those used for IP. WB detection was then performed using horseradish peroxidase (HRP)-conjugated secondary antibodies.

### 4.11. Dopamine and Homovanillic Acid (HVA) Measurement

Mouse dopamine (DA) ELISA Kit (MyBiosource, San Diego, CA, USA, Cat No. MBS269234) and mouse homovanillic acid (HVA) ELISA kit (MyBiosource, Cat No. MBS1601074) were performed on brain tissue lysates as described above according to manufacturers’ protocol.

### 4.12. Statistical and Data Analysis

All statistical analysis was performed using GraphPad Prism version 5.0 (GraphPad software, Inc, San Diego, CA, USA). One-way analysis of variance (ANOVA) (and nonparametric or mixed) tests were used in comparison of means of multiple groups. Two-tailed Student’s *t* test (and nonparametric tests) was used in comparison of mean of two groups. Shapiro–Wilk test was used for checking normality before performing statistic tests. Asterisks denote actual *p*-value significances (* <0.05, ** <0.01, *** <0.001 and **** <0.0001), and *n* is the number of animals or the number of independent experiments (cell culture) per group. Unless otherwise indicated, data are expressed as Mean ± SD.

## 5. Conclusions

In conclusion, USP13 seems to be a major player in alpha-synuclein-associated pathology, including alpha-synuclein accumulation, dopamine transmission, and motor and behavioral defects. USPs are implicated in many movement disorders, including alpha-synucleinopathies, and BK50118-C is a potential therapeutic agent.

## Data Availability

This study includes no data deposited in external repositories.

## Supplementary Materials

The following supporting information can be downloaded at: https://www.mdpi.com/article/10.3390/ijms23158131/s1
