# Supplementary material for: Inhibition of Ubiquitin-Specific Protease-13 Improves Behavioral Performance in Alpha-Synuclein Expressing Mice
Source: Int J Mol Sci. 2022 Jul 23;23(15):8131. doi: 10.3390/ijms23158131 (PMC9330474; doi:10.3390/ijms23158131)
Supplement: Supplementary file 1 [file ijms-23-08131-s001.zip › ijms-1804857-supplementary.pptx]

## Slide 1
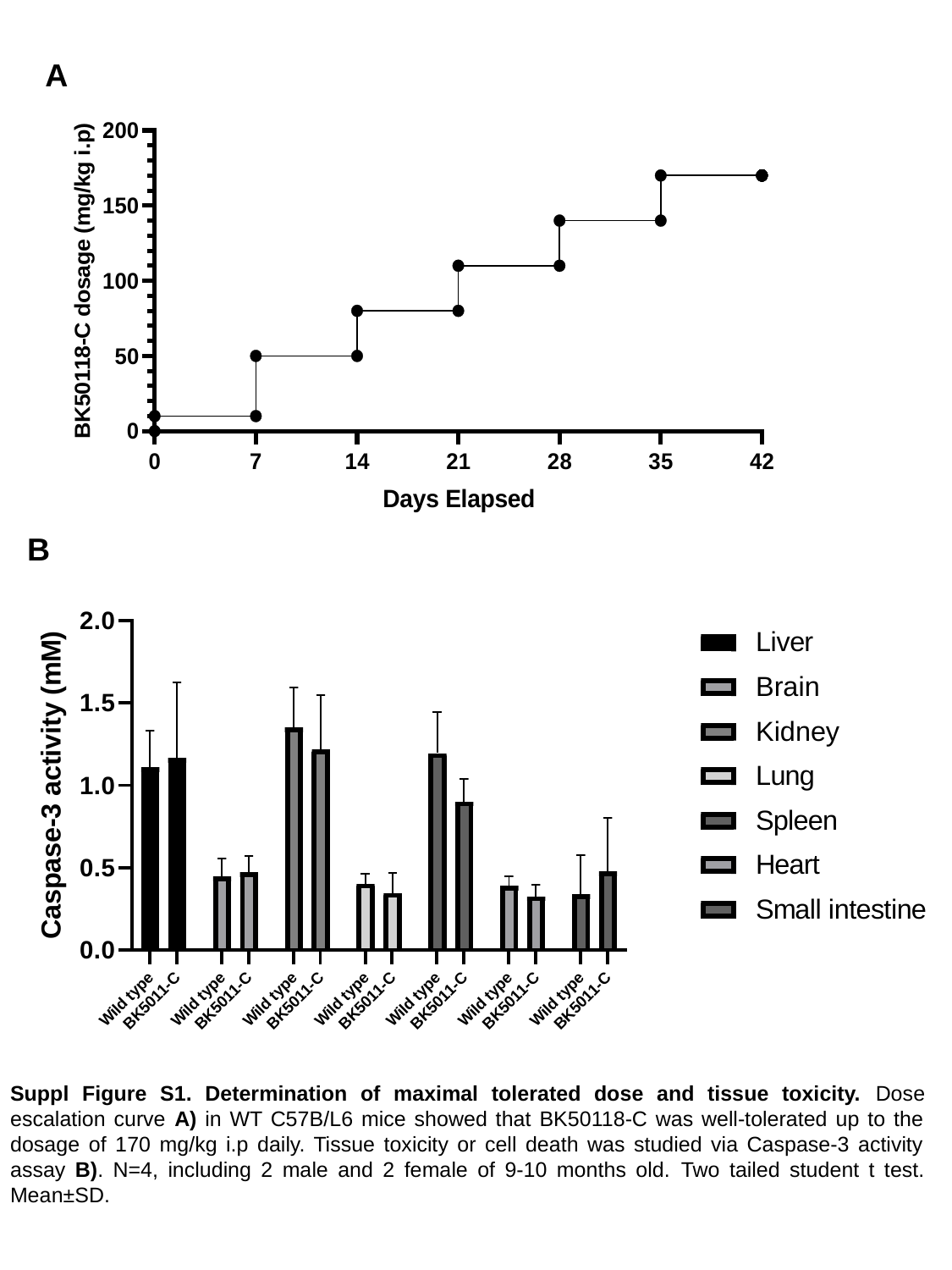

A
B
Suppl Figure S1. Determination of maximal tolerated dose and tissue toxicity. Dose escalation curve A) in WT C57B/L6 mice showed that BK50118-C was well-tolerated up to the dosage of 170 mg/kg i.p daily. Tissue toxicity or cell death was studied via Caspase-3 activity assay B). N=4, including 2 male and 2 female of 9-10 months old. Two tailed student t test. Mean±SD.

## Slide 2
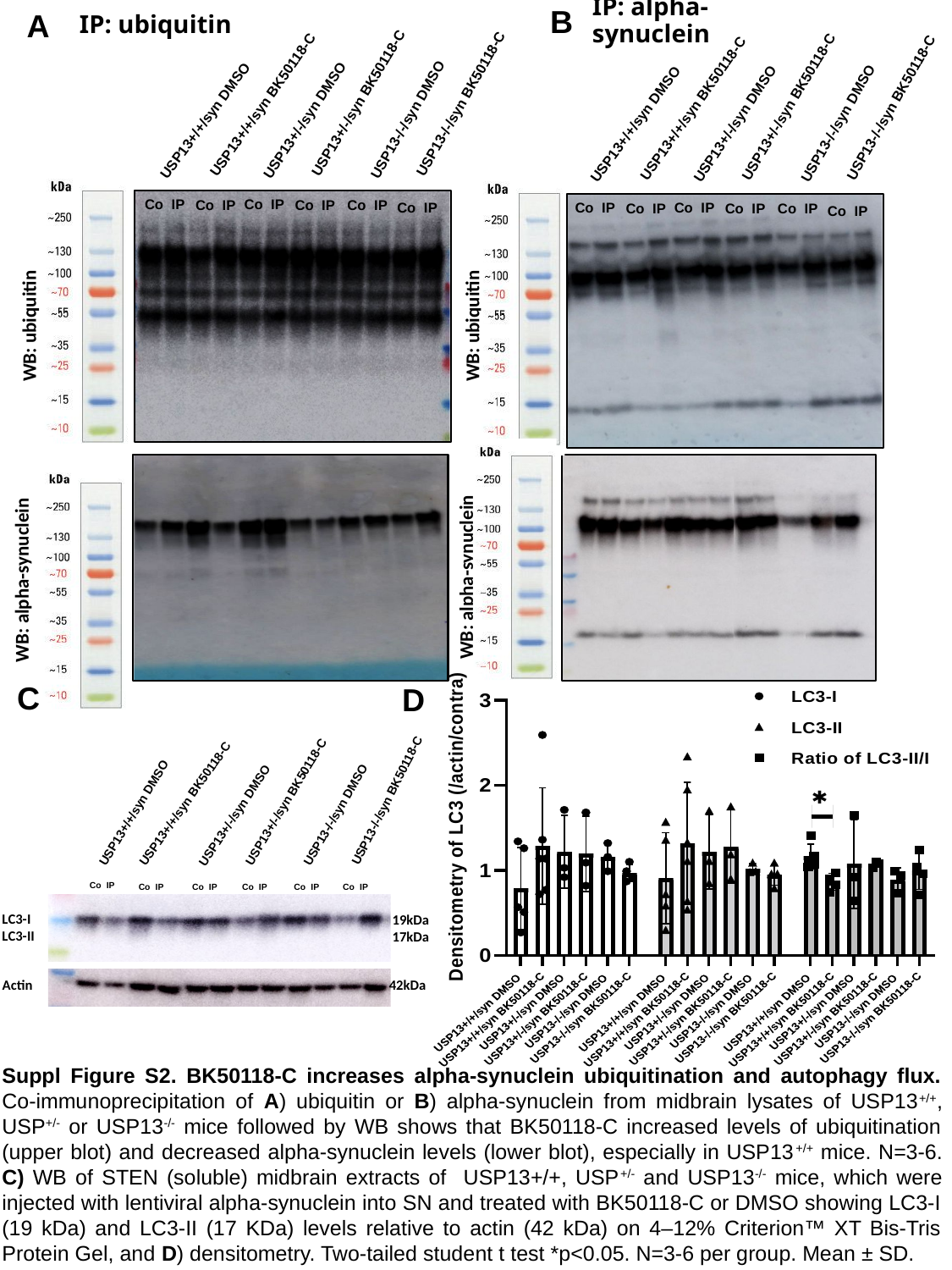

IP: alpha-synuclein
B
A
USP13+/-/syn BK50118-C
USP13-/-/syn BK50118-C
USP13+/+/syn BK50118-C
USP13-/-/syn DMSO
USP13+/-/syn DMSO
USP13+/+/syn DMSO
Co IP
Co IP
Co IP
Co IP
Co IP
Co IP
USP13+/-/syn BK50118-C
USP13-/-/syn BK50118-C
USP13+/+/syn BK50118-C
USP13-/-/syn DMSO
USP13+/-/syn DMSO
USP13+/+/syn DMSO
Co IP
Co IP
Co IP
Co IP
Co IP
Co IP
# IP: ubiquitin
WB: ubiquitin
WB: ubiquitin
WB: alpha-synuclein
WB: alpha-synuclein
C
D
USP13-/-/syn BK50118-C
USP13+/-/syn BK50118-C
USP13+/+/syn BK50118-C
USP13+/+/syn DMSO
USP13-/-/syn DMSO
USP13+/-/syn DMSO
Co IP
LC3-I
LC3-II
19kDa
17kDa
Actin
42kDa
Co IP
Co IP
Co IP
Co IP
Co IP
Suppl Figure S2. BK50118-C increases alpha-synuclein ubiquitination and autophagy flux. Co-immunoprecipitation of A) ubiquitin or B) alpha-synuclein from midbrain lysates of USP13+/+, USP+/- or USP13-/- mice followed by WB shows that BK50118-C increased levels of ubiquitination (upper blot) and decreased alpha-synuclein levels (lower blot), especially in USP13+/+ mice. N=3-6. C) WB of STEN (soluble) midbrain extracts of USP13+/+, USP+/- and USP13-/- mice, which were injected with lentiviral alpha-synuclein into SN and treated with BK50118-C or DMSO showing LC3-I (19 kDa) and LC3-II (17 KDa) levels relative to actin (42 kDa) on 4–12% Criterion™ XT Bis-Tris Protein Gel, and D) densitometry. Two-tailed student t test *p<0.05. N=3-6 per group. Mean ± SD.
